# Influence of Schneiderian Membrane Perforation on Implant Survival Rate: Systematic Review and Meta-Analysis

**DOI:** 10.3390/jcm13133751

**Published:** 2024-06-27

**Authors:** Verónica Schiavo-Di Flaviano, Sonia Egido-Moreno, Beatriz González-Navarro, Eugenio Velasco-Ortega, José López-López, Loreto Monsalve-Guil

**Affiliations:** 1 Faculty of Medicine and Health Sciences (Dentistry), University of Barcelona, L’Hospitalet de Llobregat, 08007 Barcelona, Spain; v.schiavo@ub.edu; 2 Department of Odontostomatology, Faculty of Medicine and Health Sciences (Dentistry), University of Barcelona, L’Hospitalet de Llobregat, 08007 Barcelona, Spain; beatrizgonzalez@ub.edu; 3 Oral Health and Masticatory System Group (Bellvitge Biomedical Research Institute) IDIBELL, University of Barcelona, L’Hospitalet de Llobregat, 08007 Barcelona, Spain; 4 Comprehensive Dentistry for Adults and Gerodontology, Faculty of Dentistry, University of Seville, 41004 Sevilla, Spain; evelasco@us.es (E.V.-O.); lomonsalve@hotmail.es (L.M.-G.)

**Keywords:** maxillary sinus membrane, sinus floor augmentation, dental implants, bone regeneration

## Abstract

**Background**: Maxillary sinus lift is a well-documented and accepted technique in the rehabilitation of the posterior maxilla. Schneiderian membrane perforation is the most common complication and may occur in between 7% and 56% of cases. Different materials and techniques have been described to achieve reparation of the perforation. The aim of this study was to establish whether the perforation of the Schneiderian membrane and its repair during maxillary sinus lift surgery results in a lower implant survival rate compared to those cases where the membrane has not been perforated. **Materials and methods**: A systematic review and meta-analysis of studies assessing the survival rate of implants placed in regenerated sinus using the lateral window approach, where the perforation of the Schneiderian membrane occur, was carried out. Statistical analysis was performed with Open Meta-Analyst, calculating the odds ratio of implants placed in perforated sinuses and non-perforated sinuses. **Results**: Ten articles were included in the qualitative analysis and seven articles in the quantitative analysis or meta-analysis. A total of 1224 maxillary sinus augmentation surgeries were performed without perforation of the Schneiderian membrane and 2725 implants were placed; 62 implants failed during the follow-up period with an overall survival rate of 97.7%. In 480 perforated sinuses, 1044 implants were placed, of which 30 failed; the overall survival rate was 97.1%. There were no significant differences between the implant survival rate of the implants in the two groups (OR = 0.78; CI = 0.49–2.23; *p* = 0.28 and I^2^ heterogeneity: 0%, *p* = 0.44). **Conclusions**: Schneiderian membrane perforation, as long as it is repaired, does not appear to negatively influence implant survival rate. Membrane perforation should not be considered a reason to abort the procedure or an absolute contraindication to implant placement.

## 1. Introduction

Implant rehabilitation of the posterior maxilla can be a challenge for the professional due to the pneumatization of the maxillary sinus or bone resorption after tooth loss [1]. The insufficient bone height can condition the correct placement of the implants; in these cases, it may be necessary to implement techniques that help us increase the bone availability, such as guided bone regeneration using resorbable and non-resorbable membranes, block graft, and bone distraction, among others [2,3], being that the sinus lift is one of the most-performed techniques [4].

Sinus lift is a well-documented and accepted technique in posterior maxilla rehabilitation and was introduced by Tatum H [5] and Boyne and James [6] in the 1980s. The open technique consists of preparing a window on the lateral aspect of the maxillary sinus and subsequently releasing and elevating the Schneiderian membrane, allowing the space between the membrane and the bone to be filled with graft material [7].

Schneiderian membrane perforation is the most frequent complication during a sinus lift and can occur in between 7% and 56% of cases [8,9,10,11,12]. There are various factors, both anatomical and technical, that influence the perforation, including the thickness of the membrane, previous sinus pathology, the presence of sinus septa, existing perforations, the design of the osteotomy, inadequate management of the membrane, and/or the surgeons experience [12,13,14,15]. Different techniques have been described to solve perforations, such as membrane suturing and the use of collagen membranes and fibrin glues, among others [11,13,16,17].

**Figure 1:**
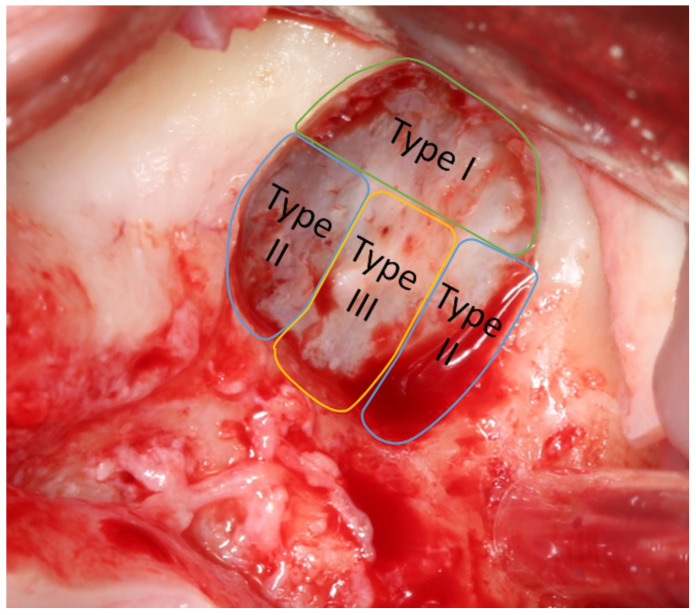
Diagram created by the authors to illustrate the classification of the perforations of the maxillary sinus membrane described by Fugazzotto and Vlassis [13].

Fugazzotto and Vlassis [13] proposed a classification for perforations according to size and the difficulty of reparation. Class I perforations are those that occur in the upper part of the osteotomy; the separation of the membrane from the bone will eventually close due to membrane folding upon itself. Class II occurs close to the lateral or lower walls of the osteotomy and its treatment is more complex. Class III perforations are located right in the center of the osteotomy window and are frequently preexisting, either due to a previous traumatic dental extraction or an oroantral fistula, although they can occur during the preparation of the membrane as well; their clinical management is similar to those of class II (Figure 1).

The most frequent postoperative complications associated with sinus elevation that we can find are acute sinusitis, infection, and loss of graft material; the last of which, according to some studies, is greater in sinuses with perforated membranes. For this reason, some authors believe that there may be a relationship between perforation of the membrane during sinus lift and implant failure [8,16,18].

The aim of this systematic review and meta-analysis is to establish whether perforation of Schneiderian membrane and its repair during maxillary sinus lift results in a lower implant survival rate compared to sinus elevations where the membrane has not been perforated.

## 2. Materials and Methods

### 2.1. Data Sources and Search Criteria

The following review was written using PRISMA guidelines [19].

The PICO question that arises is: Does perforation of Schneiderian membrane cause an increased risk of implant failure? (P) Patient/Problem: perforation of Schneiderian membrane during sinus lift; (I) Intervention: Membrane repair; (C) Control: Sinus elevations without perforation of the membrane; (O) Result: decreased survival rate.

A bibliographic research was carried out on the Medline/Pubmed, Scopus and Web of Science platforms on May 2024 using the MeSH term “Sinus floor Augmentation” associated with Boolean operators AND and OR, combined with the following keywords: (“Schneiderian membrane” OR “Maxillary Sinus Membrane” OR “Sinus Augmentation” OR “Sinus Lift” OR “Sinus Floor Elevation”) AND (“Perforation Repair” OR “Schneiderian membrane perforation” OR “maxillary sinus membrane perforation”) AND (“Implant survival”).

### 2.2. Inclusion and Exclusion Criteria

Inclusion and exclusion criteria were compiled to ensure methodological consistency across all studies being included in the meta-analysis and to address potential study-level bias.

The search was limited to articles in English. The inclusion criteria were prospective and retrospective in vivo human studies. Only those articles where sinus elevation was performed using the lateral window technique with the placement of graft material were included. The sample had to include sinus elevations where perforation of the Schneiderian membrane occurred, and the repair technique used was described. Studies with a follow-up period of at least 6 months after prosthesis placement were included. Randomized and non-randomized clinical trials, cohort studies, case-control studies, and case series were accepted. Bibliographic reviews, systematic reviews and meta-analysis, clinical trials in animal models, and reports of clinical cases were excluded. Studies in which no graft material was used after sinus lift, that included elevation using the transcrestal technique, or that were associated with other bone regeneration techniques in the sample were considered not to meet the inclusion criteria, as were studies that did not specify the number of lost implants or the type of bone graft material that was used.

### 2.3. Data Extraction

All titles were analyzed to rule out irrelevant, repeated, animal model, or in vitro studies. The abstracts were then analyzed to assess the basic characteristics of the study. The publications that remained after abstract analysis were subjected to a full text study and chosen according to the inclusion and exclusion criteria.

The information extracted from the articles was: authors, year of publication, study design, number of patients included, graft material used in sinus lift, number of sinus lifts, number of sinuses with membrane perforation, material used for membrane repair, incidence of membrane perforation, number of implants placed, and number of failed implants.

### 2.4. Risk of Bias

To assess any potential risk of bias, the authors critically appraised each study by the Newcastle–Ottawa Scale. It was developed to assess the quality of nonrandomized studies. A ‘star system’ has been developed in which a study is judged on three broad perspectives: the selection of the study groups; the comparability of the groups; and the ascertainment of either the exposure or the outcome of interest for case-control or cohort studies, respectively [20].

### 2.5. Variables Studied and Statistical Analysis

The only variable studied was the survival rate of implants placed in sinuses where Schneiderian membrane perforation occurred compared to non-perforated membranes. The implants included in the study were those implants that remained present and functional during the follow-up period.

The data meta-analysis was performed with the Review Manager 5.4 to analyze the difference between implant survival rates in sinuses with perforated membranes and sinuses without perforation. The study group consisted of the sinuses where there was perforation of the Schneiderian membrane, while the control group consisted of the sinuses where there was no perforation. The analysis method used was the binary random effect and the odds ratio was calculated with a 95% confidence interval. The forest plot was made to graphically represent the results. The level of significance was established with *p* < 0.05. Heterogeneity among studies was considered statistically significant for a *p*-value < 0.05 and was interpreted as recommended by the Cochrane Handbook: 0–40% was considered unimportant, 30–60% as moderate heterogeneity, 50–90% as substantial heterogeneity, and 75–100% as considerable heterogeneity.

To evaluate the outcome between 1-stage and 2-stage implants performed in perforated membranes or in non-perforated, a subgroup analysis has been done.

## 3. Results

**Figure 2:**
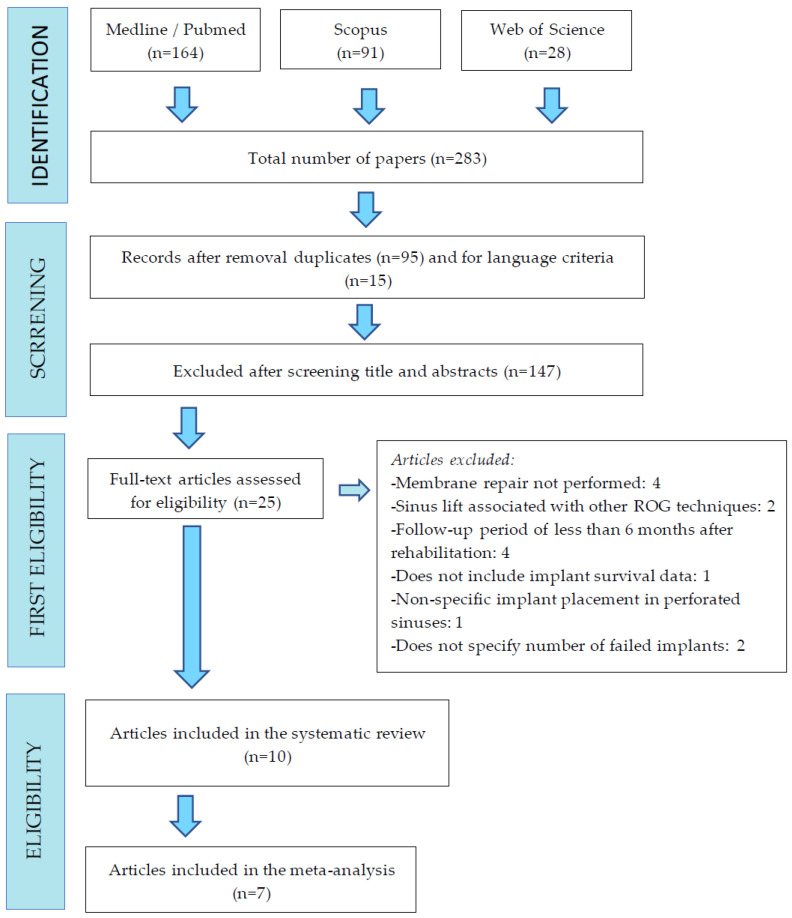
Flowchart illustrating the search strategy and selection process.

A total of 283 titles were obtained after the bibliographic research of Medline/Pubmed; in addition, 91 titles were obtained from Scopus and, finally, 28 from Web of Science. Fifteen articles were discarded because they were not written in English; 95 titles were duplicated. In the first screening of titles and abstracts, 26 articles were chosen for full-text reading. Of these 26 articles, 15 were discarded according to the inclusion and exclusion criteria. Finally, after reading the full text, 10 articles were selected to be analyzed (Figure 2).

Of the articles included in the qualitative analysis (*n* = 10), five were retrospective observational studies classified as cohort studies [10,18,21,22,23], two were case-control studies [24,25], two were case series [26,27] and one was a clinical trial [28] (Table 1). Only seven articles were included in the quantitative analysis or meta-analysis [10,21,22,23,24,25,28].

In all studies, the surgical technique used was the sinus lift with a lateral window and graft material was used to fill the sinus. Five studies used only one type of biomaterial [21,23,24,26,28] and five used between two and four different types of biomaterials [10,18,22,25,27]. In four of them, autologous bone mixed with xenograft or allograft was used [10,18,25,27]. The most widely used was xenograft [10,18,22,23,24,25,27,28], followed by alloplastic graft [21,22,26,27], autologous bone with or without combining with xenograft or allograft [10,18,25,27], and lastly, allograft [22,27]. In the cases where there was perforation of the Schneiderian membrane (635 sinuses), seven of the studies performed repair with collagen membranes [18,21,22,23,25,27,28] and two of them were associated with fibrin glues [27,28]. Demineralized human cortical bone [10], suture of the membrane with vicryl [18,23], pedicled Bichat fat ball graft [18], block graft [18], oxidized cellulose [21], palatal connective tissue graft [26], and Platelet-rich fibrin membranes (PRF) [24] were also used for repair. The mean follow-up of the patients was 25.15 months (range 6–98 months).

The total number of patients included was 1666; a total of 2229 sinus lifts were performed, and 5052 implants were placed (range between 18 and 1588 implants). The sample size regarding implant placement in articles is heterogeneous. In two articles, fewer than 50 implants were placed [24,26]; in another two, fewer than 100 implants were placed [22,27]; in three, more than 200 but fewer than 500 were placed [10,21,28]; and in two, more than 1000 were placed [18,24] (Table 2).

Schneiderian membrane perforation occurred in 635 sinuses (Range: 10–237) and the mean incidence of perforation was 29.42% (Table 2). Postoperative care was not specified in two articles [10,21]; in the remaining eight, all included antibiotic treatment, six of them were associated with non-steroidal anti-inflammatories [18,23,24,25,26,28], five with rinses with chlorhexidine 0.12–0.2% [22,23,24,25,28], and one with corticosteroids [28]. In all studies, postoperative care was the same for perforated and non-perforated sinus.

The placement of the implants was carried out simultaneously with the sinus lift or in two phases. In two articles, all the implants were placed during the same surgical procedure [18,28], and in three, they were placed during a surgery carried out between 6 and 9 months after sinus elevation [22,24,26]. In the remaining five articles [10,21,23,25,27], the implants were placed simultaneously or during a second surgery, depending on the height of the residual bone crest. Among the postoperative complications described are sinusitis [21,25,27], infection of the surgical wound [27], rhinorrhea [27], and graft necrosis [25]. Six articles did not specify whether or not there were postoperative complications [10,18,22,23,24,28], and in one of them, there were none [26] (Table 2).

The Newcastle–Ottawa scale [20] allowed to classify the case-control and cohort studies included in the systematic review as follows: two studies [18,22] scored 7 points and four studies [10,21,23,25] scored 6 points, so could be considered as low risk of bias; and one study [24] scored five points (Table 3).

Three articles were discarded for the meta-analysis: The study carried out by Hernández-Alfaro et al. [18] was discarded for not specifying the data of the control group, and the studies by Gehrke et al. [26] and Kim et al. [27] were discarded because there was no control group.

**Figure 3:**
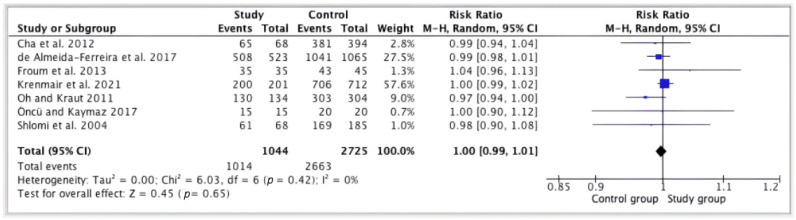
Forest plot; survival of implants in perforated versus non-perforated sinuses [10,21,22,23,24,25,28].

In total, 1224 sinus elevations were performed without perforation of the Schneiderian membrane, and 2725 implants were placed, of which 62 failed during the follow-up period, obtaining an overall survival rate of 97.7%. In 480 elevations where the membrane was perforated, 1044 implants were placed, of which 30 failed in the follow-up period, with an overall survival rate of 97%. There were no significant differences between the implant survival rates of the two groups (RR = 1.00; CI = 0.99, 1.01; *p* = 0.65); I^2^ heterogeneity was 0% (*p* = 0.42) (Figure 3).

**Figure 4:**
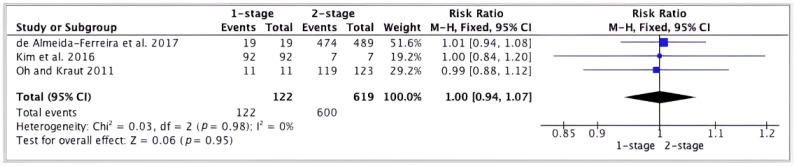
Forest plot; survival of implants in perforated sinuses of one-stage vs. two-stage implants [21,23,27].

Regarding the evaluation of the outcome between one-stage and two-stage implants. A forest plot was made to represent the difference between the results of one-stage vs. two-stage implants performed in perforated membrane cases. Three articles [21,23,27] were chosen because, in their studies, the data of one-stage and two-stage were provided. The results were not statistically significant (RR = 1.00; CI = 0.94, 1.07; *p* = 0.95); I^2^ heterogeneity was 0% (*p* = 0.98) (Figure 4).

**Figure 5:**
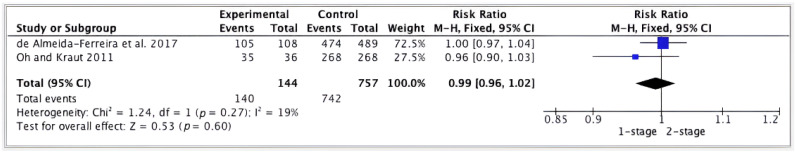
Forest plot; survival of implants in non-perforated sinuses of one-stage vs. two-stage implants [21,23].

Focusing on the evaluation of the outcome between one-stage vs. two-stage implants in non-perforated sinus membranes, a forest plot was performed to represent the difference in these results. Two studies [21,23] provided data between one-stage and two-stage implants in non-perforated sinuses. The results were not statistically significant (RR = 0.99; CI = 0.96, 1.02; *p* = 0.60) and I^2^ heterogeneity was 19% (*p* = 0.27) (Figure 5).

## 4. Discussion

Lateral sinus window elevation is a well-known and predictable technique for implant rehabilitation of the atrophic maxilla [18,22,26]. As we already remarked, the most common intraoperative complication is Schneiderian membrane perforation, with an incidence between 16% and 56% [8,11,13,15,18,28]. In this review, results were found within this range, with a perforation rate of 29.42%. Different factors have been related to an increased risk of perforation. Ardekian et al. [29] found that 85% of the perforations occurred in patients with 3 mm residual alveolar ridges vs 25% in patients with 6 mm ridges. The presence of sinus septa is found amongst 13% and 35.3% of the sinuses [30]. Septed sinuses add difficulty to surgical management of the procedure since they increase the risk of perforation; it is advisable to adapt the osteotomy and divide it into smaller sections [18]. Other factors such as the design of the window, the size of the window, the presence of mucous retention cysts, and the skills and experience of the surgeon also play a fundamental role in the appearance of complications [12,13,15].

Some authors consider that the presence of perforations in the sinus membrane signifies a contraindication to continue the procedure [8,31,32]. However, in this review it was observed that there are techniques to repair or cover the membrane perforation without having to abort the procedure, with an overall implant survival rate (ISR) of 97.1% in sinuses with perforated and repaired membranes.

In this review, the complication of Schneiderian membrane perforation was found in 635 cases and was treated using different techniques and materials for its repair. Despite that the gold standard technique for the management of perforations is not described in the literature, most of the authors reviewed used collagen membranes [18,21,22,23,27,28]. Other less common alternative materials and techniques have also been described, and according to this review, have also proven to be effective. The absorbable suture [18,23] 25 and oxidized cellulose (Surgicel^®^) [12,21] are very frequently used and easily accessible materials, which can be useful in some cases. The average thickness of the Schneiderian membrane (1.32 ± 0.87 mm) should be taken into account [33] when considering closing the perforation with suturing, since it is a difficult procedure and requires high precision from the professional.

Techniques such as block grafts [18], connective tissue [26], the use of the Bichat fat ball [18,34], or the membranes of Platelet Rich Fibrin (PRF) [24,35,36] could have a better biocompatibility due to their autologous origin. These techniques are subjected to the biological availability of the tissue, instruments and equipment, in addition to the skills of the surgeon.

Other techniques reported in the literature include demineralized human cortical bone (Lambone^®^ Pacific Coast Tissue Bank, Los Angeles, CA, USA) [10], fibrin glue (Greenplast^®^ Green cross, Gyeonggi-do, Republic of Korea) [28,37], autologous periosteum grafts [38] and amniotic membranes (Amnion-Chorion barriers, BioXclude^®^, Snoasis Medical, Denver, CO, USA) [39].

In a study conducted by Hernández-Alfaro et al. [18], with a sample of 474 sinus elevations and the placement of 1166 implants, the perforations were classified according to size and the sample was divided into three groups. Of 104 perforations, those smaller than 5 mm were the most frequent, followed by perforations between 5–10 mm and, finally, those greater than 10 mm. The implant survival rate was 97.14%, 91.89%, and 74.14%, respectively. With these results, the authors concluded that the larger the perforation, the lower the implant survival rate. The data from this review lead to the conclusion that small perforations do not significantly influence the outcome of implant treatment.

In the study carried out by de Almeida-Ferreira et al. [23] with the placement of 1588 implants in 745 sinuses, the global ISR of implants placed in sinuses with perforated membranes was 97.1% and in sinuses with non-perforated membranes it was 97.7%. Within the group of perforated membranes, the perforations were divided similarly to the study by Hernández-Alfaro et al. [18]; in this study, a survival rate of 95.3% was obtained for large perforations, 97.3% for medium perforations, and 97.7% for small perforations. The ISR differences between the three groups were not statistically significant, and in the case of small perforations, it was exactly the same as in sinuses with non-perforated membranes. Schwartz-Arad et al. [9] found no relationship between membrane perforation and the presence of postoperative complications with the success of the implants. As long as the perforation of the membrane is properly treated, it will not influence the success of the implants [29]. Despite finding that the survival rate of implants in non-perforated sinus is higher than in perforated sinus, at 97.7% vs. 97,1% respectively, the difference between the two groups is not statistically significant.

Even though the difference in the inclusion criteria that allowed us to include more studies, our results confirm those reported by Diaz-Olivares et al. [40] in their systematic review and meta-analysis, where the ISR among perforated membranes was 97.71%, vs. 98.88% in the non-perforated group.

Al-Moraissi et al. [41], in their systematic review, observed a greater survival rate, with statistical significance, between the perforated membranes group (89.65%) and the non-perforated membranes group (97.51%). Nevertheless, it should be highlighted that the present systematic review only included the results of procedures using the lateral window approach, while the previous authors included both the lateral and crestal approaches.

Many authors hypothesize that the lower survival rate of implants in perforated sinuses is due to the displacement of biomaterial particles that can trigger an acute or chronic sinusitis, producing a reabsorption of the graft and compromising the prognosis of implants [16,18,42]. Amongst the complications of the procedure sinusitis, infection of the wound, rhinorrhea, and graft necrosis were described. In the study conducted by Krennmair et al. [25] a higher incidence of sinusitis was found in perforated membranes, similar to the results reported from Oh and Kraut [21].

Concerning the survival rate of one-stage or two-stage implants in perforated or intact Scheneridan membrane, non-statistically significant differences were found. The literature found similar survival rates for implants placed in one-stage or two-stage [23]. Hence, immediate and sinus lifting in one-stage could provide us benefits like the reduction of the number of surgeries or a decrease in the treatment time [28].

Regardless of the fact that sinus elevation is a highly predictable procedure [43] and the most used biomaterial for its elevation has been beta tricalcium phosphate [44], regarding the placement of implants in regenerated sinuses, i.e., simultaneously vs. delayed, it is still controversial. If the residual ridge is greater than 5 mm, implant stability is generally achieved [18]. However, if it is less than 5 mm, it can be considered insufficient mechanical support, advising a delayed placement [45]. Cha et al. [28] did not find statistically significant differences in the success rate of implants placed in residual alveolar ridges of >5 mm (97.33%) vs. <5 mm (95.50%) (*p* = 0.3135). In reference to this issue, some authors defend that the regenerative result of sinus lift is compromised by perforation of the membrane and, therefore, the simultaneous placement of implants should not be performed [10]. Other authors consider that perforation of the membrane should not be considered a contraindication for simultaneous implant placement [18].

If we focus on the quality of regenerated bone, Testori et al. [46] obtained between 22% and 26% of vital bone in sinuses with perforated membranes, using collagen membranes that allowed the containment of the graft. Similar results were obtained by Froum et al. [22] when conducting a histological and histomorphometric study, with a higher percentage of vital bone formed in sinuses with perforated membranes (26.3 ± 6.3%) than in sinuses with non-perforated membranes (19.1 ± 13.7%). A possible explanation for these results could be that the membrane placed between the Schneiderian membrane and the graft acts as a barrier, preventing soft tissue migration, or it performs better containment and immobilization of the graft material, facilitating revascularization [22].

The limitations of this study were the non-inclusion of other variables, such as the design and treatment of the implant surface, the type of graft material, the surgical skills of the surgeon, and the patient’s habits, which may influence the survival rate of the implants.

## 5. Conclusions

Schneiderian membrane perforation is a common complication in sinus elevations and can occur in up to 30.5% of cases. There are different materials and techniques that allow the membrane to be repaired and the perforation to be covered successfully. Schneiderian membrane perforation, as long as it is repaired, does not appear to negatively influence the implant survival rate. Considering the above points, membrane perforation should not be considered a reason to abort the procedure nor as an absolute contraindication to implant placement.

## Figures and Tables

**Table 1:** Summary of the 10 articles reviewed. CM = Collagen Membrane. CTG = Connective Tissue Graft. FG = Fibrin glue. PRF = Platelet rich fibrin. * Autologous cortical bone obtained from the lateral window, symphysis block, or mandibular retromolar space.

Author, Year	Type of Study	Graft Material	Schneiderian Membrane Repair	Follow-Up
Shlomi et al., 2004 [10]	Cohort study	Autologous	Lambone^®^ demineralized human cortical bone	24 months
		Autologous + xenograft (Bio-Oss^®^)		
Hernández-Alfaro et al., 2007 [18]	Cohort study	Autologous + xenograft (Bio-Oss^®^)	Suture with Vicryl^®^	6 months
			CM (Bio-Gide^®^)	
		Autologous block	Human cortical bone *	
			Bichat fat ball graft	
Oh and Kraut 2011 [21]	Cohort study	Alloplastic (hydroxyapatite + calcium carbonate Proosteon^®^)	Oxidized cellulose (Surgicel^®^)	12 months
			CM (Ace Surgical^®^)	
Gehrke et al., 2012 [26]	Case series	Alloplastic (hydroxyapatite NanoBone^®^)	CTG from palate	12 months
Cha et al., 2012 [28]	Clinical trial	Xenograft (Bio-Oss^®^)	CM (Bio-Gide^®^) + FG (Greenplast^®^)	36–98 months
Froum et al., 2013 [22]	Cohort study	Xenograft Bio-Oss^®^)	CM (Bio-Gide^®^/CollaTape^®^)	6–32 months
		Alloplastic (BoneCeramic^®^)		
		Allograft (Puros^®^)		
Kim et al., 2016 [27]	Case series	Autologous	CM (Rapiderm^®^, Ossguide^®^, CollaTape^®^, Bio-Gide^®^) + fibrin	6–60 months
		Xenograft (Bio-Oss^®^)		
		Allograft (Ora-Graft^®^)		
		Alloplastic (Novosis^®^)		
		Autologous + xenograft/Allograft		
Öncü and Kaymaz. 2017 [24]	Case Control	Xenograft (Apatos^®^)	PRF membrane	6–12 months
De Almeida-Ferreira et al., 2017 [23]	Cohort study	Xenograft (Bio-Oss^®^)	Suture with Vicryl	24 months
			CM (CollaCote^®^)	
Krennmair et al., 2022 [25]	Case Control	Autologous + xenograft (Bio-Oss^®^)	CM (Bio-Gide^®^)	12 months

**Table 2:** Incidence of Schneiderian membrane perforation, postoperative measurements, implant placement, and complications. Pat = Patients. Imp = Implants. PSM = Perforation of Schneiderian Membrane. NE = Not Specified. ATB = Antibiotic. NSAIDs = Non-steroidal analgesics. CLH = Chlorhexidine.

Author, Year	N.° Pat.	N.° sinus	N.° Imp	N.° PSM	Perforation Incidence (%)	Postoperative Care	Implant Placement		Postoperative Complications	
Shlomi et al., 2004 [10]	63	73	253	20	28%	NE	Simultaneous		NE	
							2 phases 4–6 months			
Hernández-Alfaro et al., 2007 [18]	338	474	1166	104	21.9%	ATB/7 days NSAID	Simultaneous		NE	
Oh and Kraut 2011 [21]	128	175	438	60	34%	NE	Simultaneous		4 sinusitis	
							2 phases 8 months			
Gehrke et al., 2012 [26]	10	10	18	10	NE	ATB/7 days NSAID	2 phases 6 months		No complications	
Cha et al., 2012 [28]	161	217	462	35	16.1%	ATB/5 days NSAID/5 days Corticosteroids CLH 0.2%	Simultaneous		NE	
Froum et al., 2013 [22]	23	40	80	15	37%	ATB/7–10 days CLH 0.12%	2 phases 6–9 months		NE	
Kim et al., 2016 [27]	41	41	99	41	NE	ATB/3 days	Simultaneous		8 sinusitis	
							2 phases 4–6 months		6 local infections	
									10 rhinorrheas	
Öncü and Kaymaz. 2017 [24]	16	20	35	10	NE	ATB/14 days NSAID/14 days CLH 0.12%	2 phases 6–8 months		NE	
de Almeida-Ferreira et al., 2017 [23]	531	745	1588	237	31.8%	ATB/10 days NSAID CLH 0.12%	Simultaneous		NE	
							2 phases 6–10 months			
Krennmair et al., 2022 [25]	355	434	913	103	23.80%	NSAID, CLH, ATB/8 days, decongestant spray	Simultaneous		Sinusitis	
									10	4
							2 phases	NE	Graft necrosis	
									7	1
Total	1666	2229	5052	635						
Media					29.42%					

**Table 3:** Quality assessment of included studies using the Newcastle–Ottawa scale.

Case-Control	Selection	Comparability	Exposure	Score (0–9)
**Öncü and Kaymaz** [24]	★★★	★	★	5
**Krennmair et al.** [25]	★★★	★	★★	6
**Cohort Studies**	**Selection**	**Comparability**	**Outcome**	**Score (0–9)**
**De Almeida-Ferreira et al.** [23]	★★★	★	★★	6
**Oh et al.** [21]	★★★	★	★★	6
**Hernández-Alfaro et al.** [18]	★★★★	★	★★	7
**Froum et al.** [22]	★★★★	★	★★	7
**Shlomi et al.** [10]	★★★	★	★★	6

## Data Availability

The authors can provide details of the research upon request by letter commenting on their needs.
